# Cultivation of Nordic *Chlorococcum* sp. in anaerobic digestion effluent: Effects of CO_2_ concentration and reactor configuration

**DOI:** 10.1038/s41598-026-51126-5

**Published:** 2026-04-28

**Authors:** Ghasem Mohammadkhani, Amir Mahboubi, Christiane Funk, Päivi Ylitervo

**Affiliations:** 1 Swedish Centre for Resource Recovery, University of Borås, Borås, 50190 SE Sweden; 2 Department of Chemistry, Umeå University, Umeå, 901 87 SE Sweden

**Keywords:** CO_2_ concentration, reactor configuration, *Chlorococcum* sp., anaerobic digestion effluent, Biotechnology, Ecology, Ecology, Environmental sciences

## Abstract

The increasing discharge of untreated wastewater poses risks to ecosystems and public health, necessitating sustainable treatment strategies. Anaerobic digestion (AD) of sewage sludge offers several benefits including waste-volume reduction and sludge stabilization. However, it produces nutrient-rich effluents, requiring further treatment. Microalgae can remove nutrients while generating valuable biomass. This study aimed to evaluate the effect of CO_2_ concentration and reactor configuration on the performance of *Chlorococcum* sp. cultivated in AD effluent of municipal sewage sludge. Four CO_2_ levels (0.04, 3, 6, and 9%) was tested and 6% CO_2_ yielded the highest biomass (0.98 g L^− 1^) and CO_2_ fixation rate (162 mg L^− 1^ d^− 1^), while maintaining ammonium and phosphorous removal comparable to aeration with 3 and 9% CO_2_. This concentration was used in ALR, BC, and BC with carriers. The highest nutrient removal was achieved in BC, with 37.61% NH_4_⁺-N and 25.87% phosphorus reduction, whereas growth in ALR reached the highest cell density (81 × 10^6^ cells mL^− 1^) in 9 days. Biomass composition was stable across reactors, with similar protein, carbohydrate, or fatty acid methyl esters content. These findings demonstrate that the Nordic *Chlorococcum* sp. grown in AD effluent can remove NH_4_⁺-N and phosphorus across a wide CO_2_ range (0.04–9%). Culturing in ALR is the preferred option for rapid growth. However, BC offered better nutrient removal and higher biomass production but required longer cultivation than ALR.

## Introduction

The increasing release of untreated wastewater due to urban growth and industrial activities endangers aquatic environments and human health, primarily through contamination with heavy metals, organic compounds, and pathogens^1^. As freshwater resources decline and global water consumption is projected to rise by 55% by 2050^2^, the recycling and reuse of wastewater has become urgent. Untreated effluents lead to eutrophication and waterborne diseases, making effective wastewater treatment essential for safeguarding ecosystems and ensuring sustainable water use^1,3^. Anaerobic digestion (AD) of sewage sludge is a widely adopted method that can address this issue by transforming organic materials into biogas and removing harmful pathogens. This not only reduces health hazards associated with sludge disposal but also yields an effluent rich in nutrients^4^. However, additional treatment is necessary for this effluent to comply with discharge regulations, mainly for ammonium nitrogen^5^.

Microalgae offer an effective biological approach to wastewater treatment by assimilating nitrogen and phosphorus^6^, reducing contaminants such as heavy metals and chemical oxygen demand (COD)^7^. Moreover, microalgae generate biomass that can be used for various valuable applications such as biofuels, biofertilizers, biostimulants^8,9^, biopolymers^10^, or biochar^11^. Due to their minimal energy requirements and strong potential for resource recovery, microalgae-based technologies are both cost-effective and environmentally friendly. Their integration with AD effluent enhances nutrient removal and supports a circular bioeconomy^12^.

CO_2_ is an essential carbon source for photosynthesis, and both microalgal growth and biomass production are strongly influenced by CO_2_ concentration. However, excessively high CO_2_ concentrations may inhibit photosynthesis and reduce growth owing to significant pH reduction, highlighting the importance of identifying optimal CO_2_ levels^13^, which are strain-dependent because each microalgal strain shows unique tolerance and performance thresholds. For example, *Chlorella vulgaris* has been reported to achieved a maximum biomass concentration of 2.12 g L^− 1^ at 10% CO_2_^14^, whereas *Chlorella pyrenoidosa* attained its highest biomass concentration of 4.3 g L^− 1^ under 5% CO_2_^15^. In another study, Harwati, Willke and Vorlop^16^ reported that *Chlorococcum* sp. demonstrated optimal growth and lipid accumulation when aerated with 6% CO_2_, but showed reduced cell density and total lipid content at 10%, indicating a threshold beyond which further CO_2_ enrichment may hinder performance. Furthermore, elevated CO_2_ levels could induce metabolic shifts, leading to increased lipid and carbohydrate accumulation^15,17^.

Another important parameter that directly affects photosynthetic efficiency and microalgal growth is CO_2_ availability for the cells, which can be constrained by suboptimal mass transfer such as short residence times of CO_2_ bubbles and limited mixing. In photobioreactors, CO_2_ is typically sparged into the system as bubbles. Bubbles size and behave affect gas distribution and transfer efficiency, ultimately impacting microalgal biomass productivity^18^. For instance, techniques such as hollow fiber membranes (HFMs) for bubble-less gas exchange have been shown to improve CO_2_ delivery and biomass accumulation^19^. The efficiency of CO_2_ mass transfer is influenced by factors such as gas distributor design, sparger orifice diameter and spacing, which can increase CO_2_ retention time in the liquid phase and improve mass transfer coefficients^20^. On the other hand, transfer of CO_2_ and O_2_ is often limited by reactor geometry and operational parameters. Additionally, optimizing gas distributor design and flow rates can enhance CO_2_ dissolution and mixing, leading to better growth and carbon fixation^20,21^. Therefore, reactor design plays a critical role in addressing these limitations. Airlift Reactor (ALR) features a regular cyclic flow pattern that enhances light conversion efficiency and photosynthetic performance, supported by a centric-tube column that promotes effective mixing and uniform light distribution, resulting in higher cell concentrations and growth rates^22,23^. In contrast, the Bubble Column Reactor (BC) shows a more random fluid motion characterized by low-​frequency circular loops^22^, which is expected to result in less efficient mixing and suboptimal nutrient and light availability, ultimately yielding lower photosynthetic productivity^23^. However, the incorporation of carriers into BC reactors significantly improves their performance by providing surfaces for microalgae attachment, thereby enhancing nutrient uptake and biomass yield^24^. Additionally, they enhance gas-liquid mass transfer, increasing CO_2_ utilization and fixation rates, which further boosts microalgal growth^24,25^. They can also increase the CO_2_ residence time in the liquid phase to enhance the CO_2_ removal efficiency^26^.

Given the importance of optimizing CO_2_ concentration and reactor configuration to enhance microalgal performance, a systematic evaluation of these parameters is essential. Reactor design not only influences gas transfer and mixing but also determines nutrient uptake and biomass characteristics. Building on these insights, this study first evaluated four different CO_2_ supplementation levels (0.04, 3, 6, and 9%) to identify the optimal range for nutrient removal and microalgae growth of Nordic *Chlorococcum* sp. The selected supplementation level was then used for microalgae cultivation in three different photobioreactor designs (ALR, BC, and BC with carriers) to assess the impact of reactor design on nutrient and CO_2_ removal efficiency, biomass production, and biomass composition. The microalgae strain was the Nordic strain *Chlorococcum* sp. which has not previously been evaluated for the growth performance in anaerobic digestion effluent derived from municipal sewage sludge. Finally, this research directly advances several United Nations Sustainable Development Goals (SDGs). It contributes to SDG 13 (Climate Action) and SDG 14 (Life Below Water) by mitigating greenhouse gas emissions and reducing the risk of eutrophication. It also supports SDG 6 (Clean Water and Sanitation) by nutrient removal from AD effluent.

## Materials and methods

## Microalgae medium

Borås Energy and Environment (Borås, Sweden) supplied the liquid fraction from AD of municipal sewage sludge. The initial effluent pH was 8.1–8.3. It was first filtered using a 10 μm pore-size filter paper (Ahlstrom-Munksjo Munktell, Thermo Fisher Scientific, Sweden) and then diluted with Milli-Q water to adjust the ammonium nitrogen concentration to approximately 240 mg L⁻¹, based on the optimal level identified in our previous study^27^. The diluted effluent was subsequently autoclaved, which raised the pH to 8.8–9.1, and was used as the cultivation medium for microalgae without any further pH adjustment. The nutrient and elemental composition of the filtered AD effluent is presented in Table 1, which was kept at -20 °C until the experiment.

**Table 1:** Nutrient and elemental composition of growth medium on day 0 of cultivation.

NH_4_^+^-*N*	Concentration (mg L^− 1^)		Concentration (ppm)
	237.91 ± 11.11	Mg	4.85 ± 0.19
NO_3_-N	2.14 ± 0.23	Fe	2.14 ± 0.23
NO_2_-N	0.05 ± 0.00	Na	78.5 ± 1.50
PO_4_^−3^	9.28 ± 0.56	K	24.85 ± 0.22
Total P	4.14 ± 0.10	Cu	0.15 ± 0.01
sCOD ^a^	535.36 ± 19.25	Co	0.06 ± 0.01

^a^ Soluble chemical oxygen demand.

### Algal cultivation

The Nordic microalgal strain *Chlorococcum* sp. (MC-1) was originally isolated from a freshwater lake in Bäckhammar, located in west-central Sweden^28^. To prepare the pre-culture, *Chlorococcum* sp. was grown in sterile BG-11 medium in 500 mL Erlenmeyer flasks, with a working volume of 170 ml, sealed with cotton plugs on an open-air shaker (New Brunswick, UK) at 120 rpm at 25 °C, and exposed to continuous white light at an intensity of 80 µmol m^− 2^ s^− 1^. BG11 was prepared as described previously^27^, and the pH of BG11 was adjusted to 7.1 using 1 M HCL. Light intensity was monitored using an SKP 200 PAR Quantum Sensor (Skye Instruments, UK).

**Fig. 1:**
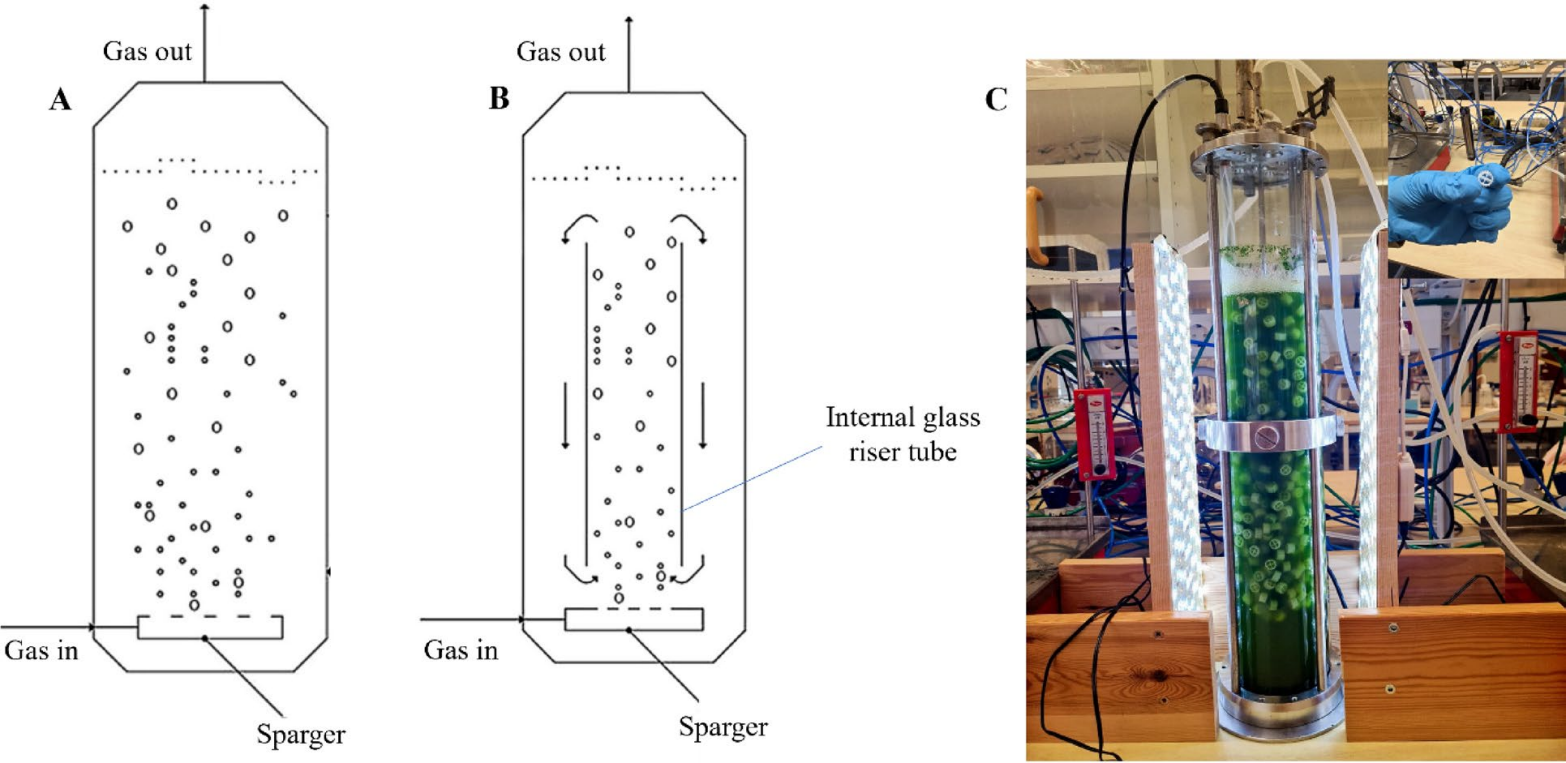
Schematic representation of the bubble column bioreactor (**A**) and airlift bioreactor (**B**). Bubble column bioreactor with carriers (**C**). Pictures A and B were adapted from^29^, with Permission from Elsevier.

A five-day old algal culture was used for inoculation to achieve an optical density (OD_750_) of 0.1 for all experimental conditions. This study was conducted in two stages. In the first stage, the effects of different CO_2_ concentrations, including 0.04 (air), 3, 6, and 9%, on microalgal growth and nutrient removal were evaluated. All cultivations during this phase were performed in BC reactors. In the second stage, the reactor configuration was investigated using the optimal CO_2_ concentration identified in stage one (6% CO_2_ mixed with air). Three reactor setups were tested: a standard BC, an ALR, and a BC with carriers (AnoxKaldnes K1-type carriers, AnoxKaldnes, Lund, Sweden) as presented in Fig. 1, and the BC with carriers was labeled as carrier in the tables and figures.

In this study, the cultivation was conducted in 4.5 L capacity glass BC bioreactors (56 cm height and 11 cm width, Belach Bioteknik AB, Skogås, Sweden). Each bioreactor was initially filled with 2.5 L of AD effluent and autoclaved at 121 °C for 20 min. After sterilization, an appropriate volume of pre-culture was added to reach OD_750_ of 0.1. The initial ammonium nitrogen concentration and working volume of each reactor were approximately 240 mg L^− 1^ and 3.5 L.

The ALR configuration was similar to the BC design but included a centered glass draft tube (40 cm height and 6 cm width). For the BC with carriers, approximately 150 g of carrier material (1 cm height, 0.5 cm width) was added, equivalent to 150 mL of liquid volume displacement.

All cultures were continuously bubbled with either air or air enriched with CO_2_ (3, 6, or 9% v/v) at a constant flow rate of 500 mL min^− 1^. Illumination was provided by continuous white light at an intensity of 100 µmol m^− 2^ s^− 1^. To prevent contamination, the gas mixtures were filtered through 0.3 μm Whatman HEPA-VENT filters (Cytiva, USA) before entering the reactor. All experiments were performed in biological duplicates over a 12-day cultivation period.​

### Microalgal growth

Every two days, a 1 mL sample was taken from each reactor to measure the optical density, cell count, and pH. The optical densities at 530, 680, and 750 nm were assessed using a visible spectrophotometer (Thermo Scientific™ GENESYS 140 Visible Spectrophotometer, Sweden). CellDrop FL (Denovix, USA, Denovix [30]) was used to determine the number of cells mL^− 1^, and the pH was assessed using a pH meter (METTLER TOLEDO, Switzerland). Samples with optical densities exceeding 1 were diluted; otherwise, the optical density was measured directly. A 10 ml sample was collected daily and centrifuged for 5 min at 3000 × g. The biomass was then washed twice with Milli-Q water to eliminate any residual components. Dry weight was measured gravimetrically [31]. At the end of the cultivation period, the cultures were harvested and washed twice with Milli-Q water. Subsequently, the biomass was freeze-dried using equipment from Labconco, USA.

### Analytical methods

The concentrations of total soluble chemical oxygen demand (sCOD), NH_4_-N, NO_3_-N, NO_2_-N, PO_4_ and total P were measured using COD 15,000, ammonium 100, Nitrite 2, standard test nitrate and nitrate 250, ortho- and total-Phosphate 15, respectively (Nanocolor^®^ Düren, Germany). A Nanocolor 500D photometer (MACHEREY-NAGEL GmbH & Co. KG, Germany) were used to assess the levels of these parameters.

The elements Fe, Mg, Ca, Na, K, Cu, and Co were quantified using Microwave Plasma-Atomic Emission Spectroscopy (MP-AES, Agilent Technologies, USA). All analyses were performed in duplicate.

To analyze the fatty acid compositions, a direct transesterification technique (D-TE) was employed, as described by Cavonius, Carlsson and Undeland [32]. Following this, the extracted fatty acid methyl ester (FAME) was subjected to gas chromatography (GC) analysis. For this purpose, a GC-FID (Perkin Elmer, Clarus 590) with a DB-23 column (Agilent, USA) was employed to examine the FAME. Nitrogen at a flow rate of 0.64 mL min^− 1^ served as the carrier gas, and the GC operated under a constant pressure of 94.45 kPa. Initially, the oven was heated to 50 °C for 2 min. The temperature then increased at a rate of 12 °C min^− 1^ until it reached 175 °C, followed by a slower ramp of 2 °C min^− 1^ until it attained 230 °C, which was held for 11 min.

Crude fat was extracted from solid samples using an ST 255 Soxtec™ extractive system (FOSS, Hillerød, Denmark). Approximately 1 g of dried material was placed in a cellulose thimble and loaded into the device, where petroleum ether served as the solvent in a three-step extraction procedure consisting of 15 min boiling, 30 min rinsing, and a 10 min solvent removal phase. The recovered extractives were collected in an aluminum cup, oven-dried overnight at 70 °C, and quantified gravimetrically. The results were reported as a percentage of the original sample weight.

To determine the protein content in the microalgal biomass, the total nitrogen content was initially assessed and then converted into protein content by applying a conversion factor of 4.78 [33]. The Element Analyzer FlashSmart CNHS (Thermo Fisher Scientific, USA) was employed to assess the total nitrogen and carbon content in microalgal biomass, with nitrogen being identified using a thermal conductivity detection system. Each tin capsule was filled with 3 to 4 mg of the sample (freeze-dried biomass) and placed in an autosampler. The combustion reactor was set to 950 °C, and the GC oven was maintained at 65 °C. Helium served as the carrier gas at a flow rate of 140 mL min^− 1^, and pure oxygen was used as the combustion gas at a flow rate of 250 mL min^− 1^. C/N ratios were determined using data from the elemental analyzer.

To assess the total carbohydrate content in microalgal biomass, a detailed standard operating procedure suggested by Chen, Gao, Song, Sommerfeld and Hu [34] was used. The carbohydrate content was determined by comparing their absorbance values at 490 nm against a glucose calibration curve created from known glucose concentrations.

Ash content was determined using a TGA/DSC 3^+^ analyzer (Mettler-Toledo, Switzerland) with a thermal profile adapted from Swedish standard (SS 187187). Samples (10 to 15 mg) were analyzed in alumina crucibles under a constant air flow of 50 mL min^− 1^. The program involved drying at 105 °C (30 min), ramping to 550 °C (60 min), holding at 550 °C (60 min), followed by cooling to ambient temperature (60 min). Ash content was calculated from the blank-corrected residual mass at the end of the isothermal hold, normalized to the initial dry mass.

### CO_2_ biofixation rate

Biomass productivity (P, mg L^− 1^ d^− 1^) was calculated from the change in biomass concentration over time, as shown in Eq. (1):

1 $$\:P=\frac{\:{X}_{1}-{X}_{0}}{{t}_{1}-{t}_{0}}\:\:\:\:\:\:\:\:\:\:\:\:\:\:\:\:\:\:\:\:\:\:\:\:\:\:\:\:\:\:$$

Where X_1_ and X_0_ represent the biomass concentration at times t_1_ and t_0_, respectively. In this study, t_0_ and t_1_ were set at day zero and 12, respectively.

The carbon dioxide biofixation rate (R_C_, mg_CO2_ L^− 1^ d^− 1^) was calculated using Eq. (2):

2 $$\:\mathrm{R}_\mathrm{C}\:=\:\mathrm{C}_\mathrm{C}\:\times\:\:\mathrm{P}\:\times\:\:\left(\frac{\:\:\mathrm{M}{CO}_{2}}{\mathrm{M}\mathrm{C}}\right)$$

Where P is the biomass productivity, and C_C_ is the carbon content of the biomass determined by elemental analysis. M_CO2_ and M_C_ are the molar mass of carbon dioxide and carbon, respectively. This approach follows established methods reported in the literature [35,36].

### Statistical analysis

All the biological experiments were conducted in duplicate. Each type of analysis, such as GC analysis, cell counting, pH measurement, OD measurement, elemental analysis, MP-AES, and test kit assessments, was performed in two replicates. Statistical analyses were carried out using Minitab^®^ 21 (Minitab Ltd., Coventry, UK). The responses were assessed using analysis of variance (ANOVA), and pairwise comparisons were performed using Tukey’s test with a 95% confidence interval. The data presented are the averages of the obtained values, and the error bars in the text, tables, and graphs indicate the standard deviations.

## Results and discussion

### Effect of CO_2_ concentration on microalgae growth

**Fig. 2:**
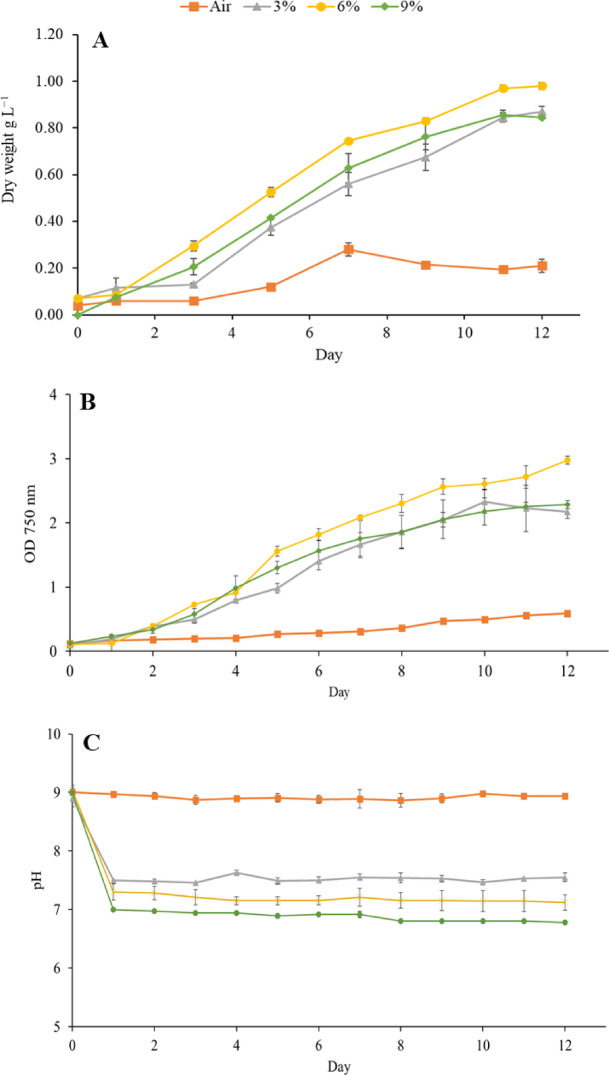
Biomass dry weight (**A**), optical densities (OD750 nm) (**B**), and pH changes (**C**) of *Chlorococcum* sp. cultures cultivated in anaerobic digestion effluents under different CO_2_ concentrations.

In the first stage of this study, *Chlorococcum* sp. was cultivated in a BC bioreactor using AD effluent from municipal sewage sludge supplemented with different concentrations of CO_2_ (0.04, 3, 6, and 9%). The biomass dry weight, optical density, and pH values of *Chlorococcum* sp. cultures under different CO_2_ concentrations with a cultivation time of 12 days are presented in Fig. 2. The pH range measured for 0.04, 3, 6, and 9% CO_2_ (average value of day 1 to 12) was 8.91 ± 0.03, 7.51 ± 0.04, 7.18 ± 0.05, 6.87 ± 0.08, respectively, indicating that increasing the CO_2_ supply from 0.04 to higher CO_2_ percentages reduced the pH values. When carbon dioxide is dissolved in water, algae cells can utilize it in photosynthesis. While active photosynthesis leads to an increased pH value in the medium, increased concentrations of CO_2_ reduce the pH as any CO_2_ not used in biomass production is transformed into carbonic acid (H_2_CO_3_). The most favorable CO_2_ concentration for growth of *Chlorococcum* sp. was 6%, contributing to the highest biomass yield of 0.98 ± 0.01 g L^− 1^. This condition also achieved significantly higher optical densities (p value of 0.026 < 0.05) in 12 days compared to the 9% concentration, and this result is in line with the data reported by Harwati, Willke and Vorlop [16]. Supplementing the culture with 6% CO_2_ resulted in a 4.6 fold increase in the final biomass concentration compared to cultivation with 0.04% CO_2_, which yielded in the lowest biomass concentration (0.21 ± 0.03 g L^− 1^) possibly due to limited photosynthesis compared to CO_2_ enriched conditions [37]. Furthermore, the medium pH was around 9 that is potentially outside the preferred pH range for *Chlorococcum* sp. This high pH was likely inhibitory due to its effect on the dissolved inorganic carbon equilibrium. This equilibrium balances dissolved CO_2_ (dominant if pH < 6.3), bicarbonate (HCO_3_^−^, dominant if 6.3 < pH < 10.3), and carbonate (CO_3_^2−^, dominant if pH > 10.3) and at pH 9 the equilibrium is dominated by bicarbonate, while the concentration of dissolved CO_2_ is negligible. Although the carbon is not yet locked as carbonate at this stage (carbonate only becomes significant above pH 10), the complete absence of free dissolved CO_2_ and the highly alkaline conditions likely created a suboptimal environment for growth, which slow photosynthesis and enhances respiration [38]. Even though some *Chlorococcum* species can tolerate pH values between 5 and 9 [39], the optimal pH for *Chlorococcum* sp. is neutral to moderately alkaline, pH 7.3 to 8.5 [16,40]. The poor growth at pH 9 aligns of the Nordic *Chlorococcum* sp. used here supports reports of its lower pH optimum [16]. Notably, exposure to 9% CO_2_ did not lead to higher biomass production than 3% or 6%, which may be caused by the lower pH values in 9% ( 6.76 < pH < 6.99) compared to 3% CO_2_ (7.46 < pH < 7.63).

### Effect of CO_2_ concentration on nutrient and CO_2_ removal

**Fig. 3:**
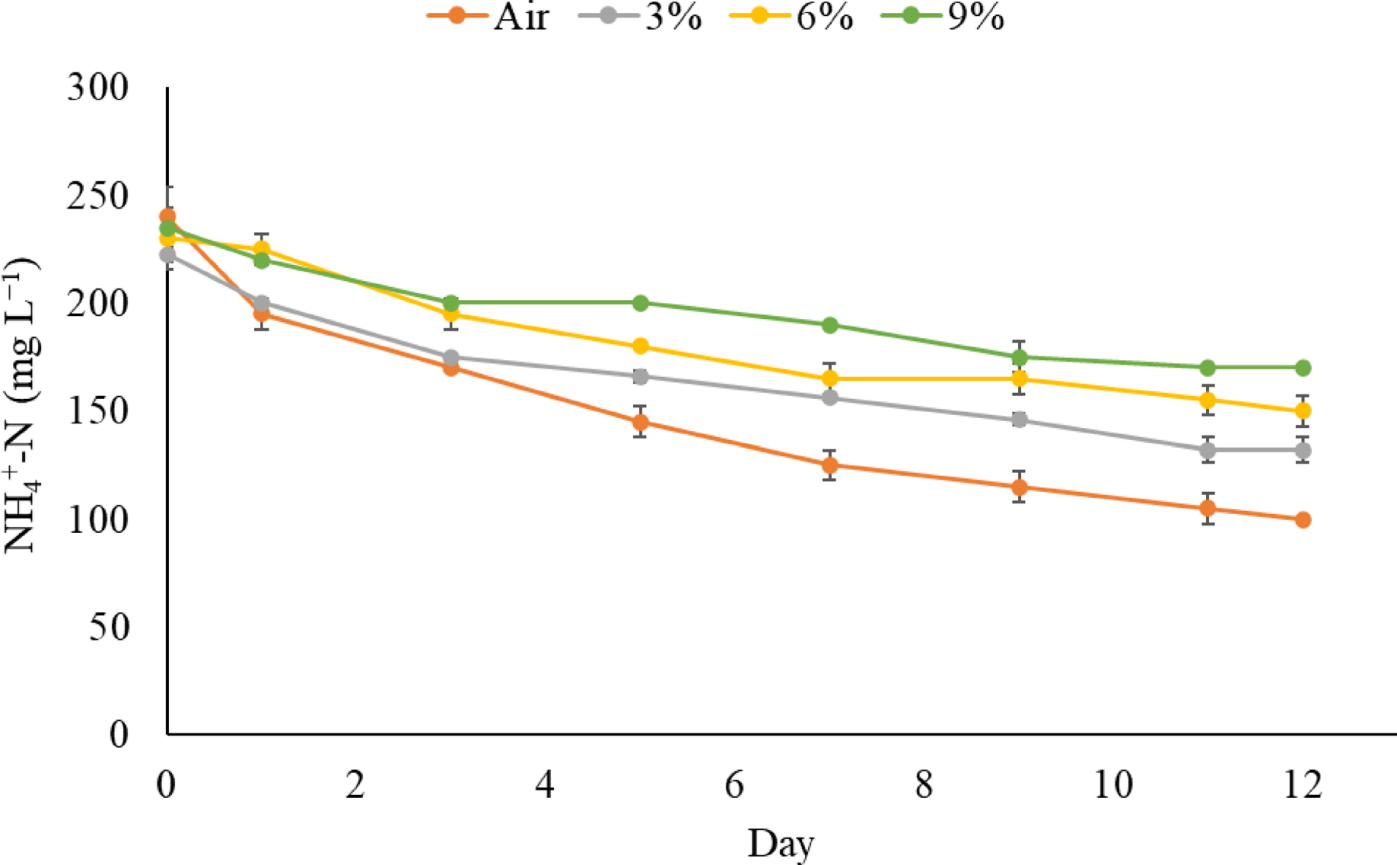
Ammonium nitrogen removal by *Chlorococcum* sp. cultures cultivated in anaerobic digestion effluents under different CO_2_ concentrations.

Figure 3 presents the ammonium nitrogen removal for different CO_2_ concentrations over 12 days. A 0.04% CO_2_ aeration resulted in significantly higher NH_4_^+^-N removal (58.04%) than those enriched with carbon dioxide, which is consistent with our previous findings. Furthermore, this condition resulted in the highest phosphorus removal rate (40.06%; Table 2).

Growth in the presence of 3% CO_2_ resulted in significantly higher NH_4_^+^-N removal efficiency than 6% or 9% CO_2_ (Table 2), while 6% CO_2_ did not result in a significantly different NH_4_^+^-N removal efficiency than 9% CO_2_. The nutrient removal efficiencies observed in this study (27–58% NH_4_^+^-N and 16–40% P removal) are within the lower to mid-range of values reported for microalgae cultivated in municipal wastewater. Higher removal efficiencies (50–85% for N and P) have been reported in large-scale tubular or raceway systems treating centrate or pre-treated municipal wastewater [41,42]. High-rate algal ponds have also achieved 76% TN and 85% TP removal when strong algae–bacteria synergy is present [43]. However, these systems typically operate at much larger volumes and longer retention times. In another study [44], *Chlorella vulgaris* cultivated under aeration rates of 1.4 to 2.3 vvm in annular airlift and bubble-column photobioreactors showed relatively low nitrogen removal, with NH_4_^+^-N consumption ranging from 16 to 19% during the initial nitrogen-sufficient stage and 30–38% during the nitrogen-reduction phase.

As illustrated in Table 2, the CO_2_ fixation rate of *Chlorococcum* sp. under CO_2_ enriched conditions was in the range of 140 to162 mg L^− 1^ d^− 1^. The highest CO_2_ fixation rate of 162 mg L^− 1^ d^− 1^ was observed with 6% CO_2_ aeration, while the lowest was noted in air aeration (27 mg L^− 1^ d^− 1^). The maximal CO_2_ fixation rate obtained under 6% CO_2_ conditions exceeded values earlier reported by the authors for *C. vulgaris* (121 mg L^− 1^ d^− 1^) or *Chlorococcum* sp. (99 mg L^− 1^ d^− 1^) under 3% CO_2_. Nonetheless, it is lower than the one reported by Nayak, Karemore and Sen [45] for *Scenedesmus* sp., which exceeded 279 mg L^− 1^ d^− 1^ under 1, 2.5, 5 or 10% CO_2_ supply.

**Table 2:** Nutrient removal efficiency, biomass productivity and CO_2_ fixation rate under different CO_2_ concentrations. Values followed by different letters within the same column are significantly different (p value < 0.05).

CO_2_	Removal efficiency (%)		Productivity	CO_2_ fixation rate
(%)	NH_4_^+^-N	P	mg _biomass_ L^− 1^ d^− 1^	mg _CO2_ L^− 1^ d^− 1^
0.04	58.04 ± 4.94 ^a^	40.06 ± 2.49 ^a^	14.58 ± 2.94 ^a^	27.05 ± 5.44 ^a^
3	40.64 ± 3.48 ^b^	25.48 ± 4.93 ^b^	66.66 ± 1.17 ^b^	146.42 ± 2.24 ^bc^
6	34.56 ± 7.09 ^c^	22.01 ± 0.43 ^b^	76.25 ± 0.58 ^b^	162.55 ± 2.28 ^b^
9	27.62 ± 2.17 ^c^	16.81 ± 3.21 ^b^	66.66 ± 0.00 ^b^	140.35 ± 5.34^c^

All cultivations were performed in duplicates in different reactors. Moreover, growth medium (without any microalgae) was tested as control samples (duplicate) to be able to exclude the effect of ammonia stripping under air aeration conditions. The initial NH_4_^+^-N concentration in the control samples of 242.5 ± 17.68 mg L^− 1^ decreased to 152.5 ± 3.54 mg L^− 1^, resulting in a removal efficiency of 36.99% ± 3.13%. pH values in the control sample ranged from 8.8 to 9.0. At this pH of 8.9 (average), the equilibrium between NH_3_ and NH_4_^+^ shifts to approximately 30.9% NH_3_ and 69.1% NH_4_^+^, suggesting that part of the ammonium removal was due to conversion to ammonia followed by ammonia stripping under air aeration conditions.

### Effect of CO_2_ concentration on biomass composition

The fatty acid composition, total fatty acid methyl esters (FAMEs), protein, carbohydrate, C/N ratio, and ash content of *Chlorococcum* sp. after growth for 12 day under different CO_2_ concentrations are shown in Table 3. The protein content (38.76–43.73%) and carbohydrate content (9.13–13.17%) in *Chlorococcum* sp. were stable under different aeration conditions, with the highest protein production of 43.73% under 0.04% CO_2_ and the highest carbohydrate production of 13.17% under 9% CO_2_. However, the total FAMEs percentage ranged from 2.88% (0.04% CO_2_) to 34.48% (9% CO_2_) of the biomass dry weight. The highest FAMEs production was achieved by 9% CO_2_ aeration, followed by 6, 3 and 0.04%, respectively.

This pattern indicates that an increased supply of CO_2_ promotes greater fatty acid production. To explain further, elevated CO_2_ enhances carbon fixation through the Calvin cycle, increasing the intracellular availability of carbon precursors such as acetyl-CoA and malonyl-CoA. These compounds are required for the cell to synthesize new fatty acids from scratch (de novo synthesis) rather than relying on modification of existing lipids. Higher CO_2_ availability can also shift cellular metabolism toward storage compounds such as lipids. Although our study did not directly quantify metabolic intermediates or enzyme activities, the observed increase in FAMEs is consistent with these established physiological responses, which is consistent with the findings of Harwati, Willke and Vorlop [16], who observed increased lipid content in *Chlorococcum* sp. at CO_2_ aeration levels of 1, 3 and 6% compared to 0.04%. Moreover, Sun, Dou, Wu, He, Wang and Chen [46] investigated the effects of aeration with CO_2_ concentrations ranging from 1% to 10% on the growth of *Chlorella sorokiniana*. Their findings showed that using 10% CO_2_ aeration significantly enhanced the lipid content compared to other tested concentrations (0.04, 1, 2 and 5%).

In this study, the primary fatty acid methyl esters of *Chlorococcum* sp. included C16:0 (palmitic acid), C18:1 (oleic acid), C18:2 (linoleic acid), and C18:3 (linolenic acid), with a smaller proportion of C16:1 (palmitoleic acid) and C18:0 (stearic acid). Other fatty acids were either not detected in the chromatograms or were present only in trace amounts below the GC-FID detection limit and therefore could not be reliably quantified. The concentration of CO_2_ aeration not only affects the quantity of total fatty acids but also affects the fatty acid profile. CO_2_ supplementation contributed to a shift from the production of dominantly polyunsaturated fatty acids (PUFAs: containing multiple carbon-carbon double bonds), specifically C18:3, to monounsaturated fatty acids (MUFAs, containing only a single double bond), such C18:1. At 0.04% CO_2_ level, the fatty acid profile was dominated by PUFAs, specifically C18:3 with 39.86%, followed by C18:2 with 20.18%. The primary MUFA, C18:1, was present at a relatively low concentration (7.61%), while the saturated fatty acid (SFA, containing no double bonds) C16:0 accounted for 19.01% of the total FAMEs. Under supplementation with CO_2_ (3, 6 and 9%), a sharp decrease in C18:3 content was observed, which dropped from nearly 40% to 13.12% (at 3% CO_2_), 14.94% (at 6% CO_2_), and 11.67% (at 9% CO_2_). This decrease in PUFAs was directly mirrored by a substantial increase in MUFA C18:1, which increased from 7.61% to a peak of 40.83% at 3% CO_2_. This result is consistent with the findings of Li, Trigani, Zuñiga, Eng, Chen, Zengler and Betenbaugh [47], who reported that *C. vulgaris* produced higher levels of C18:1 under 10% and 15% CO_2_, and increased C18:3 content when aerated with 0.04% CO_2_. Although C18:2 showed a moderate increase, C16:0 remained stable, showing no significant change across all CO_2_ concentrations. The C16:1 content, which was already low at 0.04%, decreased further to negligible levels under higher CO_2_. These data strongly suggest a metabolic shift from synthesizing building materials (membrane lipids) to storing energy reserves (storage lipids), all driven by carbon availability. Under carbon-limiting conditions (0.04% CO_2_), the organism must prioritize the use of every available carbon atom to build essential building materials, such as photosynthetic membranes (thylakoids), required to capture more energy. Therefore, the profile was dominated by PUFA C18:3, a key component of these membranes [48]. In contrast, under carbon-abundant conditions (3, 6 and 9% CO_2_), the microalgae priority switches to storing this surplus energy as triacylglycerols (TAGs) with the primary fatty acid channeled into these storage TAGs being the MUFA C18:1 [49]. This metabolic switch is explained by the C18 biosynthetic pathways. In this pathway, specific enzymes are required to create PUFAs: a delta-12 desaturase first converts C18:1 into C18:2, and then a delta-15 desaturase converts C18:2 into C18:3 [48]. The data shown in Table 3 provide evidence of the regulation of this pathway. Under high CO_2_, the sharp drop in the final product (C18:3, from 39% to 11%) and the massive accumulation of its precursor (C18:1, from 7.6% to 40%) strongly implied that the high CO_2_ concentration turned off these desaturase enzymes. This creates a metabolic bottleneck, halting the production of the membrane lipid C18:3 and causing the primary storage lipid C18:1 to accumulate.

If the algal biomass is targeted for biodiesel production, high-quality biodiesel feedstock should have a high concentration of MUFAs, such as C18:1, to ensure favorable cold-flow characteristics. At the same time, the feedstock should have low levels of PUFAs (C18:2 and C18:3) to guarantee oxidative stability [16,50]. Although PUFAs can enhance combustion properties because of their lower viscosity, they are more susceptible to oxidation, leading to reduced oxidative stability and overall fuel quality. Furthermore, elevated CO_2_ aeration promotes the synthesis of SFA C18:0, which is known for its long hydrocarbon chains that release substantial energy upon combustion, thereby enhancing the calorific value [51]. Alongside the consistently detected C16:0, these saturated lipids may enhance biodiesel properties by increasing oxidation stability and cetane number [52]. This indicates that CO_2_ aeration is required for biofuel production. As per the European Standards (EN 14214, 2004) for diesel engine quality, the amount of C18:3 and polyunsaturated acids with four or more double bonds in biodiesel is limited to a maximum of 12% and 1%, respectively, to ensure quality [53,54]. Therefore, the only condition meeting the 12% limit is 9% CO_2_, while 3 and 6% CO_2_ are very close to the limit and might be in the range with further improvement. However, if the target product is the omega-3 fatty acid C18:3, the data clearly shows that cultivation should occur under carbon-limiting conditions (0.04% CO_2_ aeration) to maximize its yield. It should also be noted that biomass produced in AD as in this study is not suitable for food applications.

**Table 3:** Fatty acid profile, total FAMEs, protein, carbohydrate, C/N ratio, and ash content of the dry biomass under different CO_2_ concentrations after 12 days of cultivation.

Fatty acid profile (%)	CO_2_ (%)			
	0.04	3	6	9
C16:0 (palmitic acid)	19.01 ± 0.17	17.93 ± 0.57	18.12 ± 0.09	17.94 ± 0.01
C16:1 (palmitoleic acid)	1.20 ± 0.40	0.38 ± 0.14	0.51 ± 0.00	0.19 ± 0.02
C18:0 (stearic acid)	0.00 ± 0.00	0.05 ± 0.01	0.14 ± 0.01	0.19 ± 0.01
C18:1 (oleic acid)	7.61 ± 0.03	40.83 ± 4.08	32.01 ± 0.32	37.82 ± 0.04
C18:2 (linoleic acid)	20.18 ± 0.29	21.2 ± 3.32	27.4 ± 0.30	26.7 ± 0.01
C18:3 (linolenic acid)	39.86 ± 0.79	13.12 ± 0.47	14.94 ± 0.01	11.67 ± 0.01
Biomass (% of dry weight)				
Total FAMEs	2.88 ± 0.06	9.79 ± 0.62	28.59 ± 2.39	34.48 ± 1.32
Protein	43.73 ± 0.33	41.58 ± 1.35	40.27 ± 0.98	38.76 ± 0.54
Carbohydrate	9.69 ± 1.00	11.44 ± 0.33	9.13 ± 1.14	13.17 ± 0.42
C/N ratio	5.62 ± 0.02	6.88 ± 0.24	6.90 ± 0.01	7.07 ± 0.17
Ash	7.92 ± 0.09	2.12 ± 0.35	2.81 ± 0.42	1.99 ± 0.19

Based on the results attained in the first stage, 6% CO_2_ concentration was chosen for the second stage of the experimental work (Sect.  3.4–3.6); this conditions resulted in highest biomass production, highest CO_2_ fixation rate among all conditions, and comparable NH_4_^+^-N and P removal compared to 3%. In the second stage different reactor designs were evaluated for optimal cultivation of *Chlorococcum* sp. in the same media.

### Effect of reactor configuration on microalgae growth

**Fig. 4:**
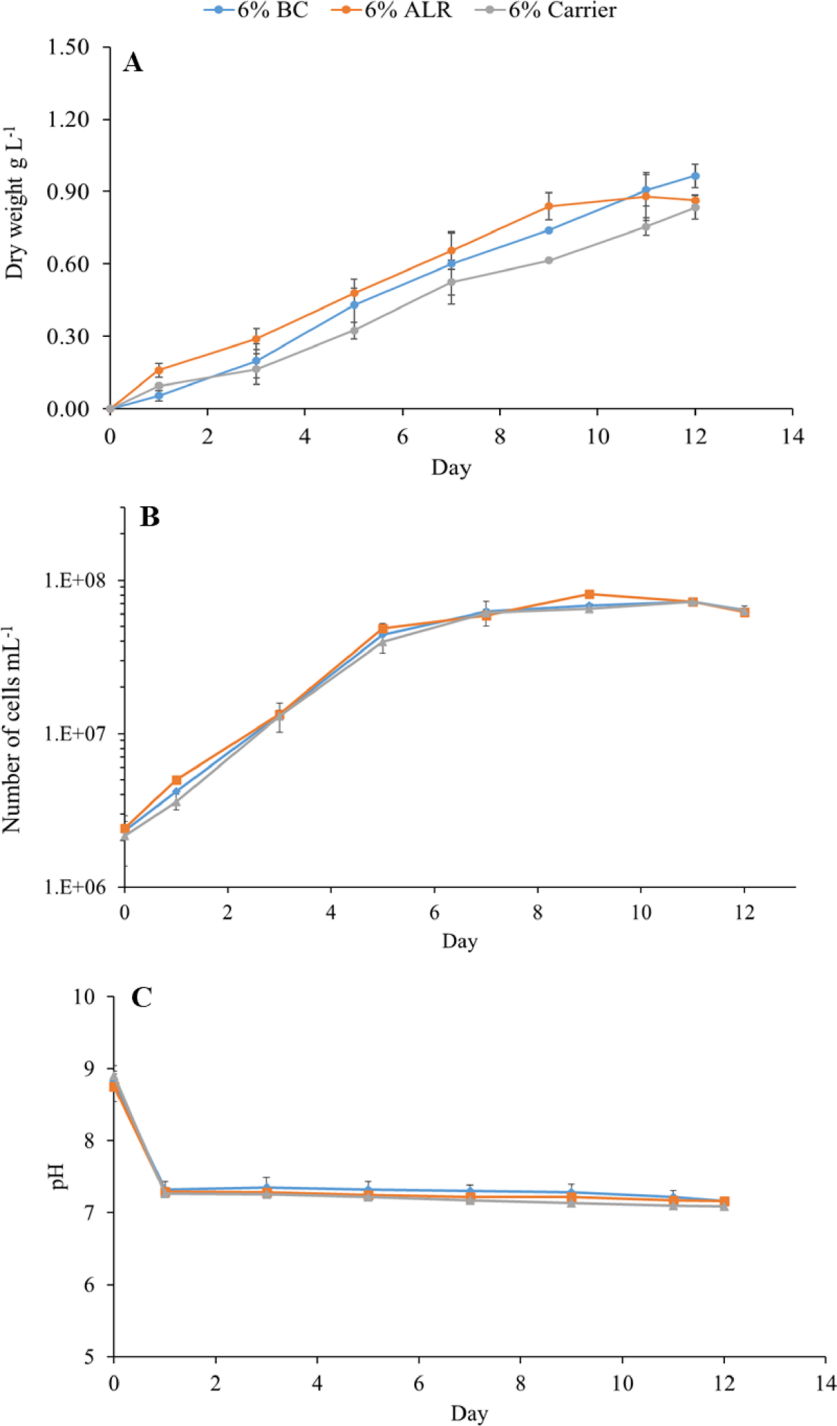
Biomass concentration (**A**), number of cells (**B**), and pH changes (**C**) of *Chlorococcum* sp. cultures cultivated in anaerobic digestion effluents using different reactor configurations.

Based on the initial assessment of various CO_2_ levels, 6% CO_2_ aeration was chosen mainly because of its superior biomass yield. This concentration was then used to evaluate the effectiveness of different reactors (BC, ALR, and BC with carriers) in terms of biomass production, nutrient removal, and biomass composition of *Chlorococcum* sp. Figure 4 presents the number of cells, biomass concentration and pH changes of *Chlorococcum* sp. using different reactor configurations for 12 days. As illustrated in Fig. 3C, from day 1 to day 12, the pH levels were stable, with average values of 7.28 ± 0.07 for BC, 7.23 ± 0.05 for ALR, and 7.18 ± 0.07 for BC with carriers. The maximum number of cells (81 × 10^6^ cells mL^− 1^) was obtained with microalgae grown in ALR on day 9, followed by BC (72.5 × 10^6^ cells mL^− 1^) and BC with carriers (72 × 10^6^ cells mL^− 1^) both on day 11 (Fig. 3B). Despite this, microalgae grown in BC yielded more biomass (0.96 ± 0.05 g L^− 1^) compared to ALR (0.86 ± 0.02 g L^− 1^) (Fig. 3A). This difference is attributed to the fact that the microalgae grown in ALR reaches the stationary phase earlier than the other reactors. This difference reflects the distinct hydrodynamic and mass-transfer characteristics of the two reactor types. The ALR provides strong internal circulation driven by the airlift loop, which enhances mixing, reduces cell sedimentation, and improves light exposure [55]. These conditions support rapid early growth (a higher biomass production than the others up to day 9), explaining why the ALR culture reached the stationary phase earlier than the BC. However, once the culture becomes dense, the ALR’s flow pattern can lead to increased light attenuation and reduced effective illuminated volume, limiting further biomass accumulation.

In contrast, the BC exhibits slower initial growth but maintains a more stable growth phase for a longer period. The BC’s larger illuminated surface area and gentler mixing can reduce photoinhibition and allow more uniform light penetration at higher biomass densities. This enables the BC to continue accumulating biomass even after the ALR culture has plateaued. Oncel and Sukan [56] reported that for *Spirulina platensis* cultivation using in BC and ALR, microalgae grown in the ALR photobioreactor produced a higher dry biomass weight concentration, achieving a maximum growth rate of 0.45 day^− 1^, whereas the microalgae grown in BC photobioreactor only reached a growth rate of 0.33 day^− 1^. It has also been noted that *Nannochloropsis* sp. achieved a higher cell concentration, resulting in improved growth when cultivated using an ALR compared to a BC, as the central tube in the ALR system provides a distinct flow pattern, superior light distribution, and more efficient mixing [23]. However, Uyar, Ali and Uyar [57] reported that *Chlorella sorokiniana* performed best in a BC photobioreactor when compared with a stirred tank and an ALR system. In their study, the BC equipped with a microporous sparger generated much smaller bubbles, resulting in significantly higher volumetric mass transfer compared to the ALR and stirred tank reactors, and ultimately superior growth (highest specific growth rate and biomass productivity).

### Effect of reactor configuration on nutrient and CO_2_ removal

**Fig. 5:**
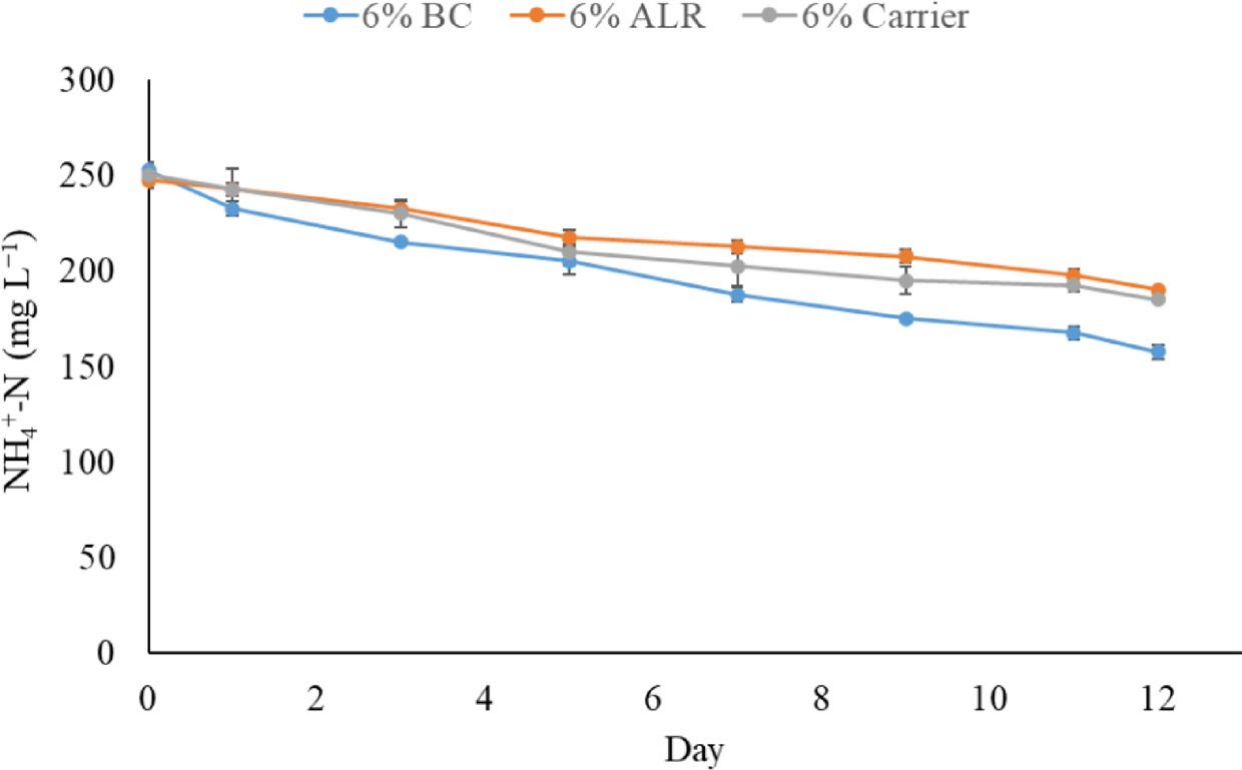
Ammonium nitrogen removal by *Chlorococcum* sp. cultures cultivated in anaerobic digestion effluents using different reactor configurations.

Figure 5 illustrates the ammonium nitrogen removal for different reactor configurations over 12 days. Cultures cultivated in BC achieved better performance in ammonium removal during the whole cultivation period, while microalgae grown in ALR and BC with carrier showed comparable results. Table 4 shows the removal efficiency of NH_4_^+^-N and P, biomass productivity, and CO_2_ fixation rate of *Chlorococcum* sp. grown in different reactor configurations.

The microalgae grown in BC reactor achieved the highest removal rates for NH_4_^+^-N and phosphorus, with 37.61% and 25.87%, respectively. Microalgae grown in ALR and BC containing carriers showed comparable performances in NH_4_^+^-N and phosphorus removal, with no statistically significant differences observed. The biomass productivity ranged from 65 to 77.08 mg L^− 1^ d^− 1^, while the CO_2_ fixation rate was between 132.45 and 155.49 mg CO_2_ L^− 1^ d^− 1^. While both parameters led to comparable results across all configurations, BC with carriers reactor showed slightly higher CO_2_ fixation rate (155.49 mg _CO2_ L^− 1^ d^− 1^). This may be attributed to the presence of carriers, which could impede the release of CO_2_ bubbles by prolonging their residence time in the liquid phase.

**Table 4:** Nutrient removal efficiency, biomass productivity, and CO_2_ fixation rates using different reactor configurations. Values followed by different letters within the same column are significantly different (p value < 0.05).

CO_2_ (%)	Removal efficiency (%)		Productivity	CO_2_ fixation rate
Reactors	NH_4_^+^-N	P	mg _biomass_ L^− 1^ d^− 1^	mg _CO2_ L^− 1^ d^− 1^
BC	37.61 ± 2.27 ^a^	25.87 ± 0.48 ^a^	65.00 ± 3.54 ^a^	132.45 ± 5.18 ^a^
ALR	23.22 ± 1.10 ^b^	15.69 ± 2.18 ^b^	67.5 ± 4.71 ^a^	135.58 ± 12.4 ^a^
Carrier	25.97 ± 2.09 ^b^	16.79 ± 2.94 ^b^	77.08 ± 5.3 ^a^	155.49 ± 11.89 ^a^

### Effect of reactor configuration on biomass composition

The fatty acid composition, total FAMEs, protein, carbohydrate, C/N ratio, ash and crude fat content of *Chlorococcum* sp. biomass after growth for 12 days using different reactors are presented in Table 5. The contents of individual fatty acids, total FAMEs, carbohydrate, protein content, and C/N ratio were comparable across all configurations, indicating that the reactor design had no significant effect on these parameters. The finding that key biochemical components remained stable across all reactor configurations demonstrates metabolic robustness in *Chlorococcum* sp., and this stability is industrially advantageous. This demonstrates that the core metabolism of *Chlorococcum* sp. is not affected by the differing hydrodynamic and gas transfer environments, which is highly advantageous for industrial scale-up, as it allows reactor selection based on cost and productivity rather than concerns over product quality. Moreover, microalgae grown in BC produced significantly higher crude fat (1.98 ± 0.08) compared to other reactor configurations. However, a huge difference between crude fat and total FAMEs content is clear, as shown in Table 5. This discrepancy arises from the extraction methods used. Crude fat was quantified using standard Soxtec extraction with petroleum ether applied directly to intact, dried biomass. Because this method does not include any cell-disruption step, the solvent cannot effectively penetrate the thick cell wall of *Chlorococcum* sp. As a result, only a small fraction of easily extractable or free lipids, approximately 2%, is recovered. In contrast, the FAMEs analysis involves transesterification, which chemically converts all saponifiable lipids (including membrane-bound and intracellular lipids) into measurable fatty acid methyl esters. Therefore, the total FAMEs value reflects the full lipid pool, whereas the crude fat value represents only the solvent-accessible portion.

In comparison with previous studies, it was expected that the presence of carriers would enhance microalgal performance, as reported in previous studies where carriers improved nutrient uptake and overall biomass productivity [24,26]. For example, Serrano-Blanco, Zan, Harvey and Velasquez-Orta [25] observed a 2.8-fold increase in cell productivity when cultivating *Tetradesmus obliquus* with suspended carriers. However, in our study, the inclusion of carriers did not lead to measurable improvements in biochemical composition or total FAMEs production by *Chlorococcum* sp. This contrast suggests that the positive effects of carriers are species- and system-dependent, and it might be offset by the light shading effect caused by the carriers.

**Table 5:** Fatty acids profile, total FAMEs, protein, carbohydrate, C/N ratio, ash and crude fat content of the biomass using different reactor designs.

Fatty acid profile (%)	Different reactor configurations (6% CO_2_)		
	BC	ALR	Carrier
C16:0 (palmitic acid)	18.11 ± 0.55	18.69 ± 1.33	18.49 ± 0.00
C16:1 (palmitoleic acid)	0.92 ± 0.11	0.87 ± 0.02	0.77 ± 0.14
C18:0 (stearic acid)	0.11 ± 0.00	0.13 ± 0.03	0.11 ± 0.03
C18:1 (oleic acid)	36.79 ± 1.02	34.22 ± 1.29	33.74 ± 0.35
C18:2 (linoleic acid)	25.79 ± 1.78	26.98 ± 0.12	26.93 ± 0.70
C183 (linolenic acid)	12.47 ± 0.26	13.07 ± 0.06	13.79 ± 0.54
Biomass (% of dry weight)			
Total FAMEs	31.49 ± 0.13	30.44 ± 0.92	30.69 ± 1.35
Protein	39.19 ± 0.67	39.91 ± 0.33	39.94 ± 0.43
Carbohydrate	9.12 ± 0.64	9.98 ± 0.78	7.64 ± 0.50
C/N ratio	6.78 ± 0.01	6.55 ± 0.19	6.57 ± 0.02
Crude fat	1.98 ± 0.08	1.44 ± 0.50	1.64 ± 0.08
Ash	2.88 ± 0.35	2.69 ± 0.53	2.72 ± 0.012

While microalgae-based systems are promising for reducing reliance on non-renewable resources, several factors still constrain their large-scale industrial application. High production costs, limited culture stability, susceptibility to inhibition, and the energy-intensive nature of harvesting remain major bottlenecks. In addition, the rigid algal cell wall often necessitates costly pretreatment steps, and regulatory uncertainties further complicate commercialization. Addressing these challenges is essential for enabling the effective scale-up of microalgal technologies and realizing their full contribution to a sustainable bioeconomy.

## Conclusion

This study investigated the ability of the Nordic microalgal strain *Chlorococcum* sp. to grow and remove nutrients from municipal sewage sludge AD effluent using different CO_2_ concentrations or reactor configurations. The results demonstrated that *Chlorococcum* sp. cultivated under 6% CO_2_ aeration supported the highest biomass production, while maintaining nutrient removal comparable to 3% and 9%. Furthermore, elevated CO_2_ levels promoted fatty acids accumulation, with total FAMEs content ranging from 2.88% to 34.48% of dry biomass. The dominant fatty acids identified were C16:0, C18:1, C18:2, and C18:3. Moreover, CO_2_ enriched aeration shifted the fatty acid profile from C18:3 toward C18:1, which may improve biodiesel quality. Reactor configurations influenced growth dynamics and nutrient removal. Growth in ALR achieved the highest cell density (81 × 10⁶ cells mL⁻¹), while cultivation in BC yielded a greater biomass concentration (0.96 ± 0.05 g L⁻¹) and highest NH_4_^+^-N and P removal (37.61% and 25.87%, respectively). Protein, carbohydrate, and FAMEs content of the microalgae remained statistically consistent independent of the reactor types, indicating that reactor choice primarily affects growth kinetics and nutrient removal rather than biochemical composition. These findings underscore the importance of optimizing CO_2_ levels and reactor design to maximize microalgal growth and achieve desired algal biomass composition for the intended application.

## Data Availability

The data that supports the findings of this study will be made available upon request.

## Declarations

### Declarations of generative AI use

Generative AI tools (Microsoft Copilot and Gemini 3 Flash) were used to improve grammar, clarity, and readability during the preparation of this manuscript. The authors take full responsibility for the content of the published article.
